# High quality draft genome sequence of *Streptomyces* sp. strain AW19M42 isolated from a sea squirt in Northern Norway

**DOI:** 10.4056/sigs.5038901

**Published:** 2014-03-01

**Authors:** Gro Elin Kjæreng Bjerga, Erik Hjerde, Concetta De Santi, Adele Kim Williamson, Arne Oskar Smalås, Nils Peder Willassen, Bjørn Altermark

**Affiliations:** 1 Norstruct, Department of Chemistry, Faculty of Science and Technology, University of Tromsø, Norway; 2 Institute of Protein Biochemistry, National Research Council, Naples, Italy

**Keywords:** Bioprospecting, enzymes, metabolites, *Streptomyces*, *Actinobacteria*

## Abstract

Here we report the 8 Mb high quality draft genome of *Streptomyces* sp. strain AW19M42, together with specific properties of the organism and the generation, annotation and analysis of its genome sequence. The genome encodes 7,727 putative open reading frames, of which 6,400 could be assigned with COG categories. Also, 62 tRNA genes and 8 rRNA operons were identified. The genome harbors several gene clusters involved in the production of secondary metabolites. Functional screening of the isolate was positive for several enzymatic activities, and some candidate genes coding for those activities are listed in this report. We find that this isolate shows biotechnological potential and is an interesting target for bioprospecting.

## Introduction

The filamentous and Gram-positive genus *Streptomyces*, belonging to the phylum *Actinobacteria* [1], are attractive organisms for bioprospecting being the largest antibiotic-producing genus discovered in the microbial world so far [2]. These species have also been exploited for heterologous expression of a variety of secondary metabolites [3]. Additionally, these species harbor genes coding for enzymes that can be applicable in industry and biotechnology [4,5].

Since the first, complete *Streptomyces* genome was published [6], a number of strains isolated from terrestrial environments have been reported [7-11]. Genomic investigations on *Streptomyces* from marine sources have, however, just recently begun [12-16].

Here, we present the draft genome sequence of *Streptomyces* sp. strain AW19M42 isolated from a marine source, together with the description of genome properties and annotation. Results from functional enzyme screening of the bacterium are also reported.

## Classification and features

The *Streptomyces* sp. strain AW19M42 was identified in a biota sample collected from the internal organs of a sea squirt (class *Ascidiacea*, subphylum *Tunicate*, phylum *Chordata*). The tunicate was isolated using an Agassiz trawl at a depth of 77m in Hellmofjorden, in the sub-Arctic region of Norway (Table 1). The trawling was done during a research cruise with R/V Jan Mayen in April 2010.

**Table 1:** Classification and general features of *Streptomyces* sp. strain AW19M42 according to the MIGS recommendations [17]

**MIGS ID**	**Property**	**Term**	**Evidence code**
		Domain *Bacteria*	TAS [18]
		Phylum *Actinobacteria*	TAS [1]
		Class *Actinobacteria*	TAS [19]
		Subclass *Actinobacteridae*	TAS [19,20]
	Current classification	Order *Actinomycetales*	TAS [19-22]
		Suborder *Streptomycineae*	TAS [19,20]
		Family *Streptomycetaceae*	TAS [19,20,22-24]
		Genus *Streptomyces*	TAS [22,24-27]
		Species *Streptomyces* sp.	NAS
		Strain AW19M42	IDA
	Gram stain	Gram positive	NDA
	Cell shape	Branched mycelia	NDA
	Motility	Dispersion of spores	NDA
	Sporulation	Sporulating	NDA
	Temperature range	Range not determined, grows at 15°C and 28°C	IDA
MIGS-6.3	Salinity	Not determined, but survives 50% natural sea water	IDA
MIGS-22	Oxygen requirements	Aerobic	NDA
	Carbon source	Not reported	
	Energy source	Not reported	
MIGS-6	Habitat	Inner organs of sea squirt	IDA
MIGS-15	Biotic relationship	Free-living	IDA
MIGS-14	Pathogenicity	Non-pathogenic	NDA
	Biosafety level	1	
MIGS-4	Geographic location	Hellmofjorden, Norway	IDA
MIGS-5	Sample collection time	April 2010	IDA
MIGS-4.1	Latitude	N67 49.24316	IDA
MIGS-4.2	Longitude	E16 28.99465	IDA
MIGS-4.3	Depth	77.35 m	IDA

Evidence codes - IDA: Inferred from Direct Assay (first time in publication); TAS: Traceable Author Statement (i.e., a direct report exists in the literature); NAS: Non-traceable Author Statement (i.e., not directly observed for the living, isolated sample, but based on a generally accepted property for the species, or anecdotal evidence). These evidence codes are from of the Gene Ontology project [28]. If the evidence code is IDA, then the property was directly observed for a live isolate by one of the authors or an expert or mentioned in the acknowledgements.

**Figure 1:**
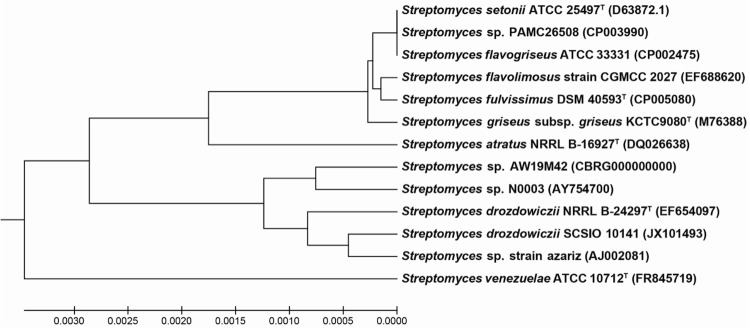
Phylogenetic tree indicating the phylogenetic relationship of *Streptomyces* sp. strain AW19M42 relative to other *Streptomyces* species. The phylogenetic tree was made by comparing the 16S rDNA sequence of the *Streptomyces* sp. strain AW19M42 to the closest related sequences from both validated type strains and unidentified isolates. *S. venezuelea* is used as outgroup. All positions containing gaps and missing data were eliminated. There were a total of 1,389 positions in the final dataset. The bar shows the number of base substitutions per site.

The bacterium was isolated during four weeks of incubation at 4-15°C on humic acid containing agar media that is selective for growth of actinomycetes [29,30]. For isolation and nucleic acid extraction the bacterium was cultivated in autoclaved media containing 0.1% (w/v) malt extract, 0.1% (v/v) glycerol, 0.1% (w/v) peptone, 0.1% (w/v) yeast extract, 2% (w/v) agar in 50% (v/v) natural sea water and 50% (v/v) distilled water, pH 8.2 [29]. The gene encoding16S rRNA was amplified by using two universal primers, 27F (5′-AGAGTTTGATCCTGGCTCAG) and 1492R (5′-GGTTACCTTGTTACGACTT) [31], in a standard *Taq* polymerase driven PCR (VWR) on crude genomic DNA prepared by using InstaGene Matrix (BioRad). Following PCR purification by PureLink PCR Purification (Invitrogen), sequencing was carried out with the BigDye terminator kit version 3.1 (Applied Biosystems) and a universal 515F primer (5′-GTGCCAGCMGCCGCGGTAA) [32]. Using the 16S rRNA sequence data in a homology search by BLAST [33] indicated that the isolate belonged to the *Streptomyces* genus, among the *Streptomycetaceae* family of *Actinobacteria*. A phylogenetic tree was reconstructed from the 16S rRNA gene sequence together with other *Streptomyces* homologues (Figure 1) using the MEGA 5.10 software suit [34]. The evolutionary history was inferred using the UPGMA method [35] and the evolutionary distances were computed using the Maximum Composite Likelihood method [36]. The phylogenetic analysis confirmed that the isolate AW19M42 belongs to the genus *Streptomyces*. The closest neighbor with a reported, complete genome sequence is *Streptomyces griseus subsp. griseus* [7], however, the phylogenetic tree indicates that the *Streptomyces* sp. strain AW19M42 isolate belongs to a closely related but separate clade. Draft genomes have not been reported for this clade previously.

## Genome sequencing and annotation

The organism was selected for genome sequencing on the basis of its phylogenetic position. The genome project is part of a Norwegian bioprospecting project called *Molecules for the Future* (MARZymes) which aims to search Arctic and sub-Arctic regions for marine bacterial isolates that might serve as producers of novel secondary metabolites and enzymes. High quality genomic DNA for sequencing was isolated with the GenElute Bacterial Genomic DNA Kit (Sigma) according to the protocol for extraction of nucleic acids from gram positive bacteria. A 700 bp paired-end library was prepared and sequenced using the HiSeq 2000 (Illumina) paired-end technology (Table 2). This generated 13.94 million paired-end reads that were assembled into 670 contigs larger than 500 bp using the CLC Genomics Workbench 5.0 software package [37]. Gene prediction was performed using Glimmer 3 [38] and gene functions were annotated using an in-house genome annotation pipeline.

**Table 2:** Genome sequencing project information

**MIGS ID**	**Property**	**Term**
MIGS-31	Finishing quality	Improved high quality draft
MIGS-28	Libraries used	One Illumina Paired-End library
MIGS-29	Sequencing platforms	Illumina HiSeq2000
MIGS-31.2	Fold coverage	350×
MIGS-30	Assemblers	CLC paired-end assembly
MIGS-32	Gene calling method	Glimmer 3
	Genbank ID	CBRG000000000
	Genbank Date of Release	September 11, 2013
	GOLD ID	Gi0070794
	Project relevance	Bioprospecting

## Genome properties

The total size of the genome is 8,008,851 bp and has a GC content of 70.57% (Table 3), similar to that of other sequenced *Streptomyces* isolates. A total of 7,727 coding DNA sequences (CDSs) were predicted (Table 3). Of these, 6,400 could be assigned to a COG number (Table 4). In addition, 62 tRNAs and 8 copies of the rRNA operons were identified.

**Table 3:** Genome statistics, including nucleotide content and gene count levels

**Attribute**	**Value**	**% of total**^a^
Genome size (bp)	8,008,851	100
DNA coding region (bp)	6,979,999	87.2
DNA G+C content (bp)	4,951,797	70.6
Total genes	7,813	n/a
rRNA operons	8	n/a
tRNA genes	62	n/a
Protein-coding genes	7,727	100
Genes assigned to COGs	6,400	82.8
Genes with signal peptides	987	12.8
Genes with transmembrane helices	1,660	21.5

^a^The total is based on either the size of the genome in base pairs or the total number of protein coding genes in the annotated genome.

**Table 4:** Number of genes associated with the 25 general COG functional categories

**Code**	**Value**	**%age**^a^	**Description**
J	264	3.4	Translation
A	1	0.0	RNA processing and modification
K	836	10.8	Transcription
L	330	4.3	Replication, recombination and repair
B	5	0.1	Chromatin structure and dynamics
D	71	0.9	Cell cycle control, mitosis and meiosis
Y	0	0.0	Nuclear structure
V	159	2.1	Defense mechanisms
T	442	5.7	Signal transduction mechanisms
M	338	4.3	Cell wall/membrane biogenesis
N	28	0.4	Cell motility
Z	6	0.1	Cytoskeleton
W	0	0.0	Extracellular structures
U	79	1.0	Intracellular trafficking and secretion
O	200	2.6	Posttranslational modification, protein turnover, chaperones
C	409	5.3	Energy production and conversion
G	665	8.6	Carbohydrate transport and metabolism
E	730	9.4	Amino acid transport and metabolism
F	123	1.6	Nucleotide transport and metabolism
H	262	3.4	Coenzyme transport and metabolism
I	330	4.3	Lipid transport and metabolism
P	435	5.6	Inorganic ion transport and metabolism
Q	417	5.4	Secondary metabolites biosynthesis, transport and catabolism
R	1,181	15.3	General function prediction only
S	465	6.0	Function unknown
-	1,327	17.2	Not in COGs

^a^The total is based on the total number of protein coding genes in the annotated genome.

All putative protein coding sequences were assigned KEGG orthology [39], and mapped onto pathways using the KEGG Automatic Annotation Server (KAAS) server [40]. The analysis revealed that *Streptomyces* sp. strain AW19M42 harbors several genes related to biosynthesis of secondary metabolites. We have identified genes that map to the streptomycin biosynthesis pathway (glucose-1-phosphate thymidylyltransferase (EC 2.7.7.24), dTDP-glucose 4,6-dehydratase (EC 4.2.1.46) and dTDP-4-dehydrorhamnose reductase (EC 1.1.1.133)). Also, several genes map to the pathways for biosynthesis of siderophore group nonribosomal peptides, biosynthesis of type II polyketide product pathway and polyketide sugar unit biosynthesis. Interestingly, two clusters, comprising five genes, both mapped to the biosynthesis of type II polyketide backbone pathway. These genes clusters comprise genes STREP_3146-3150 and STREP_4370-4374. This suite of genes may contribute to a distinct profile of secondary metabolites production.

## Insights from the Genome Sequence

**Figure 2:**
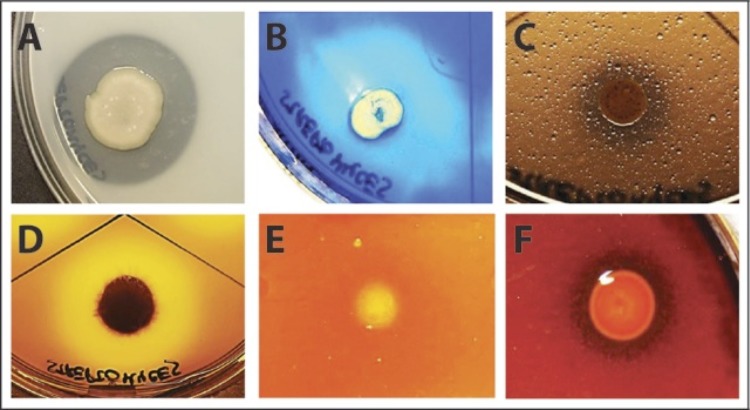
Degradation halos around colonies of *Streptomyces* sp. AW19M42 growing on agar plates supplemented with A, skim milk, B, gelatin, C, tributyrin, D, DNA, E, chitin and F, starch.

The isolate was successfully screened for lipase, caseinase, gelatinase, chitinase, amylase and DNase activities (Figure 2), by using marine broth (Difco) agar plates incubated at 20°C [41-46]. The plates were supplemented with 1% (v/v) tributyrin, 1% (w/v) skim milk, 0.4% (w/v) gelatin, 0.5% (w/v) chitin or 2% (w/v) starch, respectively (all substrates from Sigma), whereas DNase test agar (Merck) was supplemented with 0.3M NaCl, representing sea water salt concentration, before screening for DNase activity. Putative genes coding for these activities were identified in the genome based on annotation or by homology search (Table 5).

**Table 5:** Candidate genes coding for putative lipase, caseinase, gelatinase and DNase activities identified in *Streptomyces* sp. strain AW19M42 draft genome.

**Putative gene**	**Annotation**	**Size (aa)**
**Lipase**		
STREP_0737	Lipase	273
STREP_1671	Triacylglycerol lipase	266
STREP_1821	G-D-S-L family lipolytic protein	281
STREP_2698	Lipase class 2	297
STREP_2704	Triacylglycerol lipase	269
STREP_4585	Secreted hydrolase	268
STREP_5662	Lipase or acylhydrolase family protein	367
STREP_6665	Esterase/lipase	259
STREP_6850	Esterase/lipase	429
STREP_7611	Triacylglycerol lipase	366
**Gelatinase**		
STREP_5784	Peptidase M4 thermolysin	523
STREP_6038	Peptidase M4 thermolysin	680
STREP_3662	Peptidase M4 thermolysin	358
**Caseinase**		
STREP_0198	Putative secreted serine protease	361
STREP_0258	Protease	278
STREP_0974	Protease	488
STREP_1078	Serine protease	388
STREP_1313	M6 family metalloprotease domain-containing protein	398
STREP_1389	M6 family metalloprotease domain protein	1,389
STREP_2216	Putative secreted subtilisin-like serine protease	511
STREP_2239	metalloprotease	296
STREP_3135	Metalloprotease domain protein	127
STREP_3964	ATP-dependent protease La	808
STREP_3975	ATP-dependent metalloprotease FtsH	673
STREP_4000	Streptogrisin-B - Pronase enzyme B SGPB/Serine protease B	299
STREP_5179	ATP-dependent Clp protease proteolytic subunit	222
STREP_5180	ATP-dependent Clp protease, ATP-binding subunit ClpX	432
STREP_5944	Protease	527
STREP_5945	Protease	534
STREP_6196	Protease	383
STREP_6570	Protease	701
STREP_6821	Putative protease	352
STREP_7179	Serine protease	635
STREP_7580	Protease	856
**DNase**		
STREP_0436	Exodeoxyribonuclease VII, large subunit	403
STREP_0437	Exodeoxyribonuclease VII small subunit	91
STREP_1352	Exodeoxyribonuclease III Xth	268
STREP_1969	TatD-related deoxyribonuclease	1,969
STREP_2155	Deoxyribonuclease V	220
STREP_2430	Deoxyribonuclease/rho motif-related TRAM	452
STREP_4206	Deoxyribonuclease	776
STREP_6678	Probable endonuclease 4 - Endodeoxyribonuclease	275
**Chitinase**		
STREP_2729	Chitinase, glycosyl hydrolase 18 family	628
STREP_5817	Chitinase, glycosyl hydrolase 18 family	424
STREP_5513	Carbohydrate-binding CenC domain protein	577
STREP_3508	Glycoside hydrolase family protein	609
STREP_4257	Putative endochitinase	350
STREP_6187	Chitinase, glycosyl hydrolase 19 family	297
STREP_6188	Chitinase, glycosyl hydrolase 19 family	291
**Amylase**		
STREP_1696	Glycoside hydrolase starch-binding protein	573
STREP_5789	Secreted alpha-amylase	458
STREP_7405	Malto-oligosyltrehalose synthase	834
STREP_1697	Alpha-1,6-glucosidase, pullulanase-type	1,774

## Conclusion

The 8 Mb draft genome belonging to *Streptomyces* sp. strain AW19M42, originally isolated from a marine sea squirt in the sub-Arctic region of Norway has been deposited at ENA/DDBJ/GenBank under accession number CBRG000000000. The isolate was successfully screened for several enzymatic activities that are applicable in biotechnology and candidate genes coding for the enzyme activities were identified in the genome. *Streptomyces* sp. strain AW19M42 will serve as a source of functional enzymes and other bioactive chemicals in future bioprospecting projects.
